# Association between maternal depression and child stunting in Northern Ghana: a cross-sectional study

**DOI:** 10.1186/s12889-016-3558-z

**Published:** 2016-08-24

**Authors:** Anthony Wemakor, Kofi Akohene Mensah

**Affiliations:** 1 School of Allied Health Sciences, University for Development Studies, P O Box TL 1883, Tamale, N/R Ghana; 2 School of Public Health, KNUST, Kumasi, Ghana

**Keywords:** Maternal depression, Stunting, Northern Ghana, Children, Mothers

## Abstract

**Background:**

Stunting indicates failure to attain genetic potential for height and is a well-documented indicator for poor growth. Depression is common in women of reproductive age and women’s mental health problems may affect the growth of young children. We examined the association between maternal depression and stunting in mother-child pairs attending Child Welfare Clinic (CWC) in Northern Ghana.

**Methods:**

An analytical cross-sectional study was performed involving mothers (15–45 years) and their children (0–59 months) who attended CWC at Bilpeila Health Centre, Tamale, Ghana. Socio-demographic data were collected using a semi-structured questionnaire, maternal depression was measured using Centre for Epidemiological Studies Depression Screening Scale, and anthropometry was conducted on children following standard procedures. The association between maternal depression and child stunting was examined in logistic regression adjusting for potential confounders.

**Results:**

Prevalence rates of child stunting and maternal depression were estimated at 16.1 and 27.8 % respectively in Northern Ghana. Mothers with depression when compared with those without depression tended to be younger, be currently unmarried, belong to the poorest household wealth tertile, and were more likely to have low birth weight babies, so these characteristics were adjusted for. In an adjusted multivariate logistic regression model, children of depressed mothers were almost three times more likely to be stunted compared to children of non-depressed mothers (Adjusted OR = 2.48, 95 % CI 1.29–4.77, *p* = 0.0011).

**Conclusions:**

There is a high prevalence of depression among mothers in Northern Ghana which is associated with child stunting. Further studies are needed to identify the determinants of maternal depression and to examine its association with child stunting to inform nutrition programming.

**Electronic supplementary material:**

The online version of this article (doi:10.1186/s12889-016-3558-z) contains supplementary material, which is available to authorized users.

## Background

Stunting indicates a failure to achieve genetic potential for height and is a well-documented indicator for poor growth related to environmental and socio-economic circumstances [1]. Stunting is caused by long-term insufficient nutrient intake and/or frequent bouts of infections. The consequences of childhood stunting include delayed neurological development, permanent cognitive impairment, weakened immune systems [2], and a susceptibility to chronic diseases in adulthood [3]. Globally, 178 million children 5 years of age are stunted with the vast majority in South Central Asia and Sub-Saharan Africa [4]. In Ghana, the national prevalence of stunting among children under 5 years was 19 % but there are wide regional variations with 33 % of under five children stunted in the Northern Region according to the recent Demographic and Health Survey [5]. The region with the capital city, Greater Accra, has the least percentage of stunted children (10 %). Despite the several interventions being implemented for malnutrition by both Governmental and Non-Governmental Organisations in Northern Region, the rate of decline in stunting is very slow. Stunting decreased from 48.8 % in 2003 to 33.0 % in 2014 in Northern Region but came down from 32 to 14 % within the same period in a neighbouring region [5, 6]. This slow decline in stunting rate suggests that not all causative factors for malnutrition have been identified and/or are being addressed adequately.

Depression is common among women of reproductive age, and 10 to 15 % of all mothers in developed countries are estimated to suffer from depression [7]. Epidemiological studies and data on the prevalence of depression in Africa are limited but a systematic review involving 35 studies using different depression questionnaires in eight African countries found a depression prevalence of 11.3 % in pregnancy which rose to 18.3 % after birth [8]. In Ghana, two small health centre-based studies reported a depression prevalence of 10.0 % in HIV positive mothers in Eastern Region using Edinburgh Postnatal Depression Scale [9], and mothers of sick children admitted at a tertiary health care facility in Ashanti Region using the Patient Health Questionnaire [10] but in the second study, over 25 % of the mothers had scores corresponding to moderate depression. A recent large study in the Brong Ahafo Region estimated a prevalence of 9.6 % among pregnant women which reduced to 3.8 % after delivery [11]. The combination of women’s vulnerability to depression, their responsibility for childcare and the high prevalence of maternal depression in developing countries [8, 12] suggests that, maternal mental health could have a substantial influence on the growth of children in these countries.

Evidence suggests that maternal depression adversely affects psychological and intellectual development of children [13–15]. It is plausible for maternal depression to affect a child’s physical health and development through interfering with quality of emotional childcare or “mother-child attachment” [16], a known risk factor for poor growth [17], or through negatively affecting mothers’ health-promoting behaviours and child-care practices [18, 19]. Rapid physical growth and development occur in early life when infants and young children depend on the primary caregiver for their social and nutritional needs, and this makes them vulnerable to the effects of their caregivers’ mental health problems. While findings from individual studies are conflicting, two systematic reviews [20, 21] and a meta-analysis [22] summarising studies on the association between maternal depression and child undernutrition in low and middle income countries have found an association between them.

The aim of this study was to investigate the association between depression among mothers (15–45 years) and stunting in their children (0–59 months) attending Child Welfare Clinic (CWC) at Bilpiela Health Centre, Tamale, Northern Ghana.

## Methods

### Participants

An analytical cross-sectional study was performed. The study population consisted of all women (aged 15–45 years) with apparently healthy children under 5 years old seeking CWC services at the Bilpiela Health Centre or its 2 outreach posts in April–May, 2014. Mothers were briefed on the objectives of the study during the weekly CWC sessions and were enrolled to participate in the study conditional to consenting. Among the 392 mothers approached, 384 consented and were interviewed.

Bilpiela is a suburb of Tamale, the capital city of the Northern Region (one of ten administrative regions), and the third largest city by population size. Tamale is located 600 km north of Accra, the national capital city. Most residents of Tamale are moderate followers of Islam and belong to the Dagbon ethnic group.

### Measurements

Data were collected using a semi-structured questionnaire, the Centre for Epidemiologic Depression Screening (CED-S) Questionnaire and anthropometry. The administration of the questionnaire was undertaken by two final year undergraduate students of the School of Medicine and Health Sciences, UDS, Tamale. The questionnaire was administered in English in a face-to-face interview with the mothers, and for those who did not speak English it was translated and administered in Akan or Dagbani language.

### Assessment of socio-demographic characteristics

Socio-demographic information on the mothers including age, marital status, educational level, religion, and number of previous children were obtained from the respondents. Information was requested on ownership of 14 household items: mobile phone, TV, sewing machine, DVD player, satellite dish, radio, mattress, refrigerator, computer, electric fan, bicycle, motorcycycle/tricycle, animal-drawn cart, and vehicle. These household items are thought to reflect household socio-economic status. Using the 14 household items a wealth score was derived for each household using principal component analysis and the scores were ordered/classified into tertiles. The first tertile represents the lowest socio-economic group; the middle tertile the middle class and the third tertile the highest socio-economic class.

### Assessment of stunting

Anthropometric measurements were done following standard procedures [1] by two undergraduate students during CWC sessions of the Bilpiela Health Centre either at the Centre or at outreach posts. Recumbent length was measured for children below the age of 2 years and standing height for the remainder of the children up to the nearest 0.1 cm using infantometre. Children were weighed naked or wearing minimal clothing being carried by their mothers using the UNISCALE to the nearest 0.1 kg. Birth weight, sex and birthdate were recorded from the child welfare booklet and age was calculated from the date of interview and date of birth. Height-for-age, weight-for-age, and weight-for-height z-scores were derived based on the World Health Organisation (WHO) Multicentre Growth Standard [23], summarised and presented as means and standard deviations and also used to construct categorical variables. A z-score of less than −2 standard deviations was considered as stunting, underweight and wasting for height-for-age, weight-for-age and weight-for-height indices respectively [23]. Stunting was considered as the dependent variable.

### Assessment of depression

Depression status assessment was done using the Centre for Epidemiological Studies Depression (CES-D) Scale [24]. The CES-D scale comprises of 20 questions and responses and is used to assess depressive feelings and behaviours during the past week. Four questions (4, 8, 12, and 16) are statements expressing “positive thoughts” and the responses are scored from 3 to 0 with three indicating that those “thoughts” were rarely had (at most 1 day) and 0 indicating that those “thoughts” were had most days (5–7 days) of the week. The remainder of the questions are statements expressing “negative thoughts” and are reverse scored. The scores are summed up with a possible total score of 60 and a score of ≥16 is indicative of depression. Although not diagnostic, these can be used as proxy measures of depressive disorder after validation against interviews. The CED-S scale scores were added up and a 2-category variable of no depression (score < 16) and depression (≥16) was generated from the total score so that it was more clinically meaningful than a raw score. Additionally, a 4-category variable of 1. No depression (CES-D score <16), 2. Possible depression (16 ≥ CES-D score ≤ 19), 3. Depression (20 ≥ CES-D score ≤ 24), and 4. Severe depression (CES-D score ≥ 25) was constructed [25]. Ethical clearance was obtained for the study from the School of Medicine and Health Sciences, University for Development Studies, Tamale, Ghana.

### Statistical analysis

Data analysis was performed using Stata/SE version 11.0 (Stata Corp, College Station, TX, USA). Means and standard deviations were computed for continuous variables and frequencies and percentages for categorical variables. Bivariate analyses were conducted in which maternal depression status (Depressed or Not depressed) was compared with the maternal socio-demographic variables (maternal age, marital status, education level, religion, ethnicity, household wealth tertile) and child characteristics (birthweight, sex, and age) using Chi-square (*χ*^2^) for proportions and *t*-test for means. Maternal socio-demographic characteristics (age, marital status and household wealth tertile) and child characteristic (birth weight) statistically significant in the bivariate analyses were controlled for in a binary logistic regression model that assessed the association between stunting (dependent variable) and maternal depression (independent variable). In all analyses, the level of statistical significance was set at 0.05.

## Results

The socio-demographic characteristics by the entire study population and stratified by maternal depression status are presented in Table 1 below. The mean age of the mothers was 27.9 [Standard Deviation (SD) ±8.2] years and a quarter of the mothers were aged 25–29 years. The majority of the mothers had no education (64.8 %), were married (81.3 %), belonged to Dagomba ethnic group (88.8 %) and practiced Islamic religion (94.3 %). 20.6 % of the study children were low birth weight. 107 out of the 384 mothers had 20 or more scores on the CES-D screening questionnaire giving a point prevalence of maternal depression of 27.8 % (95 % CI 23.4–32.4 %), and 15.9 % had scores corresponding to possible depression. In exception of maternal age, marital status, household socio-economic wealth status and birth weight, most of the socio-demographic characteristics were evenly distributed among the depressed and non-depressed mothers. Mothers with depression tended to be younger 28.6 (SD ± 8.3) years versus 30.6 (SD ± 8.1) years (*p* = 0.020), currently unmarried 26.2 % versus 13.0 % (*p* = 0.001), belong to the poorest household wealth tertile 52.4 % versus 18.5 % (*p* < 0.001) and more likely to have low birth weight babies 32.1 % versus 11.6 % (*p* < 0.001).

**Table 1:** Socio-demographic characteristics of the study population, overall and by maternal depression status

Maternal characteristic	Total sample (*n* = 384)	No maternal depression (*n* = 216)	Maternal depression (*n* = 168)	*P*-value
	Frequency (%)/Mean (±SD)	Frequency (%)/Mean (±SD)	Frequency (%)/Mean (±SD)	
Mean age, years	27.9 (±8.2)	30.6 (±8.1)	28.6 (±8.3)	0.020
Age group, years				0.307
<20	35 (9.1)	15 (6.9)	20 (11.9)	
20–24	82 (21.4)	42 (19.4)	40 (23.8)	
25–29	76 (19.8)	41 (19.0)	35 (20.8)	
30–34	64 (16.7)	40 (18.5)	24 (14.3)	
35–39	53 (13.8)	32 (14.8)	21 (12.5)	
40+	74 (19.3)	46 (21.3)	28 (16.7)	
Education				0.819
No education	249 (64.8)	139 (64.4)	110 (65.5)	
Some education	135 (35.2)	77 (35.6)	58 (34.5)	
Marital status				0.001
Married	312 (81.3)	188 (87.0)	124 (73.8)	
Currently unmarried	72 (18.8)	28 (13.0)	44 (26.2)	
Ethnicity				0.698
Dagomba	341 (88.8)	193 (89.4)	148 (88.1)	
Other tribes	43 (11.2)	23 (10.6)	20 (11.9)	
Religion				0.868
Islam	362 (94.3)	204 (94.4)	158 (94.0)	
Other religions	22 (5.7)	12 (5.6)	10 (6.0)	
Household wealth tertile				<0.001
Poorest	128 (33.3)	40 (18.5)	88 (52.4)	
Medium	128 (33.3)	67 (31.0)	61 (36.3)	
Richest	128 (33.3)	109 (50.5)	19 (11.3)	
Child characteristic				
Mean age, months	19.5 (±16.1)	18.7 (±15.7)	20.7 (±16.5)	0.215
Age, months				0.070
0–5	84 (21.9)	56 (25.9)	28 (16.7)	
6–23	173 (45.1)	89 (41.2)	84 (50.0)	
24–59	127 (33.1)	71 (32.9)	56 (33.3)	
Sex				0.728
Female	189 (49.2)	108 (50.0)	81 (48.2)	
Male	195 (50.8)	108 (50.0)	87 (51.8)	
Mean Birth weight, kg	2.7 (±0.40)	2.9 (±0.40)	2.6 (±0.30)	<0.001
Birth weight, kg				<0.001
<2.5	79 (20.6)	25 (11.6)	54 (32.1)	
≥2.5	305 (79.4)	191 (88.4)	114 (67.9)	

Anthropometry information on the children is presented in Table 2. Both the mean anthropometric z-scores for the entire study population and those stratified by age groupings were negative indicating the children in the study were less well-nourished compared to the WHO reference standard population. More children were stunted 16.1 % than underweight 8.9 % and stunting increased with age whilst underweight was relatively stable. As expected the prevalence of wasting was lowest with a rate of 3.4 %.

**Table 2:** Anthropometric status of children

Age (months)	0–5 (*N* = 84)	6–23 (*N* = 173)	24–59 (*N* = 127)	0–59 (*N* = 384)
Z-score (Mean ± SD)				
Height-for-age	−0.43 ± 0.96	−0.86 ± 1.11	−0.82 ± 1.10	−0.75 ± 1.09
Weight-for-age	−0.57 ± 0.96	−0.82 ± 0.91	−0.71 ± 0.89	−0.73 ± 0.92
Weight-for-height	−0.33 ± 0.66	−0.48 ± 0.80	−0.37 ± 0.73	−0.41 ± 0.75
Prevalence (%, 95 % CI)				
Stunting	6.0 (0.8–11.1)	16.2 (10.6–21.7)	22.8 (15.4–30.2)	16.1 (12.4–19.8)
Underweight	7.1 (1.5–12.8)	9.2 (4.9–13.6)	9.4 (4.3–14.6)	8.9 (6.0–11.7)
Wasting	2.4 (−0.9–5.7)	4.6 (1.5–7.8)	2.4 (−0.3–5.0)	3.4 (1.6–5.2)

In the unadjusted model (Table 3), the children of depressed mothers were 3 times more likely to be stunted compared to children of psychologically sound mothers odds ratio (OR) 3.57 (95 % Confidence Interval [CI] 1.99–6.40, *p* < 0.001). Upon adjusting for socio-demographic (maternal age, marital status and household wealth tertile) and child characteristic (birth weight) that were statistically significant in bivariate analyses, the risk of stunting to children of depressed mothers decreased slightly but remain statistically significant (AOR = 2.48, 95 % CI 1.29–4.77, *p* = 0.0011).

**Table 3:** Odds ratios for stunting in children of mothers with depression compared to children of mothers without depression

Variable	Model 1 (Unadjusted) OR (95 % CI)	Model 2 (Adjusted for child characteristic) OR (95 % CI)	Model 3 (Adjusted for child and maternal characteristics) OR (95 % CI)
Maternal depression			
No	1.00	1.00	1.00
Yes	3.57* (1.99–6.40)	3.49* (1.91–6.35)	2.48* (1.29–4.77)
Birth weight (kg)			
≥2.5		1.00	1.00
<2.5		1.12 (0.58–2.15)	1.04 (0.53–2.04)
Maternal Age (years)			
15–19			1.00
20–24			1.47 (0.45–4.79)
25–29			1.90 (0.57–6.31)
30–34			1.16 (0.32–4.25)
35–39			1.80 (0.48–6.65)
40–45			1.22 (0.36–4.10)
Marital status			
Married			1.00
Not currently married			0.78 (0.36–1.71)
Household wealth tertile			
Richest			1.00
Medium			2.67 (1.09–6.59)
Poorest			3.40 (1.36–8.48)

Model 2 was adjusted for child’s birth weight

Model 3 was adjusted for child’s birth weight, maternal age, marital status and household wealth tertile

Not currently married category includes unmarried, divorced and widowed mothers

*p < 0.001

## Discussion

### Main findings

16.1 and 27.8 % of our study population have child stunting and maternal depression respectively and we found an increased risk of stunting in children of depressed mothers when compared to children of non-depressed mothers (AOR 2.48, CI 1.29–4.77) even after adjusting for potential confounders in a multivariate analysis. To our knowledge this is the first study to evaluate the impact of maternal depression on the nutritional status of Ghanaian children.

The percentages of child growth indicators measured in our study were lower compared to the regional averages of 33, 6.3 and 20 % for stunting, wasting and underweight [6], but we also found that stunting increased dramatically as the children aged suggesting problems with complementary feeding and accumulation of repeated exposures to infections. In Ghana, 22 % of children in the rural areas are stunted compared to 15 % in urban areas and rural children are more likely to experience nutritional problems compared to urban children [6]. The semi-urban nature of our study area which is in the Tamale Metropolis may explain the lower undernutrition rates measured.

There are notable disparities in socio-demographic, socio-economic and child characteristic between mothers with and without depression. Our study showed that mothers with depression were younger; currently unmarried, and were more likely to belong to the poorest household wealth tertile, or to have low birth weight babies. Data are unavailable on the determinants of maternal depression in Ghana for comparison. In Nigeria, Adewuya et al found that not currently being married increased the risk of depression in pregnant women [26]. A systematic review on low and lower middle income countries identified young age, and being unmarried as risk factors and more education as a protective factor for common perinatal mental disorders [27].

We however estimated a higher rate of depression among mothers than previously reported elsewhere in the country; 3.8–11.3 % [9–11, 28]. All the previous studies were conducted outside Northern Ghana, two involved mothers with special circumstances i.e., HIV positive mothers and mothers of sick and hospitalized children, and the other two were on women in a vitamin A trial. Three different depression screening tools and cut-offs were used in the previous studies. Clearly there is some degree of variation among the estimated prevalence rates but differences in the population studied, study design, depression screening tool and cut-off make these incomparable and these same factors may also explain some of the variation. Our rate is however closer to a prevalence rate of depression (26.6 %), measured among pregnant women at a tertiary hospital in the Ashanti Region, Ghana [29]. The high prevalence of depression measured in our study can be partly explained by the lack of formal education among the mothers; 64.8 % of mothers in our study had no form of education. Despite several health interventions, the prevalence of undernutrition in the Northern Region, Ghana has not reduced as expected. Evidence from South East Asia suggests that despite appreciable levels of food security and economic growth, child malnutrition problems are prevalent, a phenomenon referred to as the Asian “enigma” and attributed to low social status of women and/or a lack of women’s control over resources [30]. Inadequate care for children is one of the underlying causes of malnutrition, along with food insecurity and poor access to health services and unhealthy environment [31]. In this study we explored the association between maternal depression and child stunting in order to inform nutrition programming.

Our study findings agree with some studies but not others and the population studied, study design, and depression screening tools are likely to influence these associations. In Indian and Pakistan an association was detected for prenatal depression and poor child growth [32–34], but another study on British Pakistanis living in Britain did not find an association [35]. A synthesis of several of these studies concluded that there is an association between maternal depression and poor child growth and development and that this association seems to operate in low socio-economic societies only [20–22].

Maternal depression possibly promotes unhealthy lifestyles, poor health-seeking behaviours, deficient physical and emotional care and psychosocial stimulation of the infant; and these coupled with low household socio-economic status impair child growth and development [18]. Depression is known to reduce maternal interest in child rearing and caring activities [16]. In one study infants of depressed mothers were less likely to be fully immunized at 12 months compared with infants of psychological well mothers reflecting a lack of appropriate health-seeking behaviour in depressed mothers [33]. In Ghana, postnatal depression symptoms in HIV positive mothers were associated with increased diarrhoeal episodes in children [9] and in Nigeria children of depressed mothers were more likely to have poor growth, to discontinue breastfeeding, and to have more persistent diarrhoea [36].

## Strengths and limitations of study

The use of a cross-sectional study design in which both maternal depression and child stunting were assessed simultaneously does not allow for causal inference and ascertainment of the temporal order of exposure and outcome. We measured the association between maternal depression and child stunting and assumed mothers with depression are more likely to have stunted children but it could well have been poor growth in children led to depression in their mothers. We have used a validated and widely used screening questionnaire, CES-D questionnaire, in assessment of maternal depression. CES-D has not been validated in Ghana and therefore, no clinical threshold (i.e., cut-off score) has been established to distinguish between those depressed and not depressed and its reliability in this context is unknown. In addition, our study did not include a clinical diagnostic interview or ethnographic assessment that could be used as a reference standard. Some of the women we have classified as depressed may not actually have depression as CES-D questionnaire is a screening tool and not diagnostic. We failed to control for childcare support which has been found to moderate the effect of maternal psychopathology in community-based studies in low and middle income countries, such as Nigeria [26].

## Conclusions

There is a moderate level of stunting in children but the mothers of these children have a high level of depression in Northern Ghana, and these two health conditions are associated. Further research is needed to identify the determinants of maternal depression, and to verify the link between maternal depression and child stunting.

## Additional files

Additional file 1: Study dataset. (DTA 53 kb) Additional file 2: Study questionnaire. (PDF 390 kb)

## Abbreviations

AOR: Adjusted odds ratio
CED-S: Centre for Epidemiologic Depression Screening
CWC: Child Welfare Clinic
SD: Standard deviation
WHO: World Health Organisation
