# Supplementary material for: Association between maternal depression and child stunting in Northern Ghana: a cross-sectional study
Source: BMC Public Health. 2016 Aug 24;16(1):869. doi: 10.1186/s12889-016-3558-z (PMC4997709; doi:10.1186/s12889-016-3558-z)
Supplement: Additional file 2: — Study questionnaire. (PDF 390 kb) [file 12889_2016_3558_MOESM2_ESM.pdf]

UNIVERSITY FOR DEVELOPMENT STUDIES
SCHOOL OF MEDICINE AND HEALTH SCIENCES
DEPARTMENT OF COMMUNITY NUTRITION

QUESTIONNAIRE: EXPLORING THE ASSOCIATION BETWEEN MATERNAL DEPRESSION AND NUTRITIONAL STATUS OF CHILDREN (0-5 YEARS)

INFORMED CONSENT

We are final year students of the School of Medicine and Health Sciences, UDS, Tamale. We are conducting a survey on the research topic *"Exploring the association between maternal depression and nutritional status of children (0- 5 years)"* for our final year project work. We would like to ask you some few questions about yourself and your child and would very much appreciate your participation. The interview will take 10-15 minutes to complete, and whatever information provided will be kept strictly confidential. Participation is voluntary.

Questionnaire No………….

A. SOCIO-DEMOGRAPHIC CHARACTERISTICS OF MOTHER

1. **Age of mother** ……… (years)
2. **Religion** 1. Christianity 2. Islam 3. ART 4. Others
3. **Ethnicity** 1. Akan 2. Ga 3. Dagomba 4. Frafra 5. Other specify …………………………….
4. **Marital status** 1. Married 2. Single 3. Divorced 4. Widowed
5. **Educational background** 1. Primary 2. JHS/Middle School 3. Secondary/Vocational/Technical 4. Tertiary 5. No education
6. **Average income per month (GHC) ……………………………………….**
7. **Residential area ………………………………………………**
8. Please tick 1 for Yes or 0 for No against the following items in the table depending on whether the respondent's household has them.

**Item**	**1 Yes, 0 No**
Radio	
Color /black TV	
Satellite dish	
Sewing Machine	
Mattress	
Refrigerator	
DVD/VCD	
Computer	
Electric Fan	
Mobile Telephone	
Bicycle	
Motorcycle/Tricycle	
Animal-drawn cart	
Car/truck	

B. SCREENING FOR DEPRESSION

Below is a list of some ways you may have felt or behaved. Please indicate how often you have felt this way during the last week by checking the appropriate space.

1. I was bothered by things that usually don't bother me.

1. Some or little of the time (1-2 days) 2. Occasionally or a moderate amount of time (3-4 days)
3. Most or all of the time (5-7 days) 4. Rarely or none of the time (less than 1 day)

2. I did not feel like eating; my appetite was poor.

1. Some or little of the time (1-2 days) 2. Occasionally or a moderate amount of time (3-4 days)
3. Most or all of the time (5-7 days) 4. Rarely or none of the time (less than 1 day)

3. I felt that I could not shake off the blues even with help from my family or friends.

1. Some or little of the time (1-2 days) 2. Occasionally or a moderate amount of time (3-4 days)
3. Most or all of the time (5-7 days) 4. Rarely or none of the time (less than 1 day)

4. I felt I was just as good as other people.

1. Some or little of the time (1-2 day 2. Occasionally or a moderate amount of time (3-4 days)
3. Most or all of the time (5-7 days) 4. Rarely or none of the time (less than 1 day)

5. I had trouble keeping my mind on what I was doing.

1. Some or little of the time (1-2 days) 2. Occasionally or a moderate amount of time (3-4 days)
3. Most or all of the time (5-7 days) 4. Rarely or none of the time (less than 1 day)

6. I felt depressed.

1. Some or little of the time (1-2 days) 2. Occasionally or a moderate amount of time (3-4 days)
3. Most or all of the time (5-7 days) 4. Rarely or none of the time (less than 1 day)

7. I felt that everything I did was an effort.

1. Some or little of the time (1-2 days) 2. Occasionally or a moderate amount of time (3-4 days)
3. Most or all of the time (5-7 days) 4. Rarely or none of the time (less than 1 day)

8. I felt hopeful about the future.

1. Some or little of the time (1-2 days) 2. Occasionally or a moderate amount of time (3-4 days)
3. Most or all of the time (5-7 days) 4. Rarely or none of the time (less than 1 day)

9. I thought my life had been a failure.

1. Some or little of the time (1-2 days) 2. Occasionally or a moderate amount of time (3-4 days)
3. Most or all of the time (5-7 days) 4. Rarely or none of the time (less than 1 day)

10. I felt fearful.

1. Some or little of the time (1-2 days) 2. Occasionally or a moderate amount of time (3-4 days)
3. Most or all of the time (5-7 days) 4. Rarely or none of the time (less than 1 day)

11. My sleep was restless.

1. Some or little of the time (1-2 days) 2. Occasionally or a moderate amount of time (3-4 days)
3. Most or all of the time (5-7 days) 4. Rarely or none of the time (less than 1 day)

12. I was happy.

1. Some or little of the time (1-2 days) 2. Occasionally or a moderate amount of time (3-4 days)
3. Most or all of the time (5-7 days) 4. Rarely or none of the time (less than 1 day)

13. I talked less than usual.

1. Some or little of the time (1-2 days) 2. Occasionally or a moderate amount of time (3-4 days)
3. Most or all of the time (5-7 days) 4. Rarely or none of the time (less than 1 day)

14. I felt lonely.

1. Some or little of the time (1-2 days) 2. Occasionally or a moderate amount of time (3-4 days)
3. Most or all of the time (5-7 days) 4. Rarely or none of the time (less than 1 day)

15. People were unfriendly.

1. Some or little of the time (1-2 days) 2. Occasionally or a moderate amount of time (3-4 days)
3. Most or all of the time (5-7 days) 4. Rarely or none of the time (less than 1 day)

16. I enjoyed life.

1. Some or little of the time (1-2 days) 2. Occasionally or a moderate amount of time (3-4 days)
3. Most or all of the time (5-7 days) 4. Rarely or none of the time (less than 1 day)

17. I had crying spells.

1. Some or little of the time (1-2 days) 2. Occasionally or a moderate amount of time (3-4 days)
3. Most or all of the time (5-7 days) 4. Rarely or none of the time (less than 1 day).

18. I felt sad.

1. Some or little of the time (1-2 days) 2. Occasionally or a moderate amount of time (3-4 days)
3. Most or all of the time (5-7 days) 4. Rarely or none of the time (less than 1 day)

19. I felt that people disliked me.

1. Some or little of the time (1-2 days) 2. Occasionally or a moderate amount of time (3-4 days)
3. Most or all of the time (5-7 days) 4. Rarely or none of the time (less than 1 day)

20. I could not get going.

1. Some or little of the time (1-2 days) 2. Occasionally or a moderate amount of time (3-4 days)
3. Most or all of the time (5-7 days) 4. Rarely or none of the time (less than 1 day)

C. CHILD ANTHROPOMETRY

1. Date of birth (dd/mm/yyyy) ……./……./20.….
2. Age …….. (months)
3. Sex 1. Male 2. Female
4. Birth weight ………. (kg)
5. MUAC …………… (cm)
6. Current weight ……... (kg)
7. Height/length ……… (cm)
8. How height/length was measured 1. Lying 2. Standing

Thank You
